# A Fatal Waterborne Outbreak of Pesticide Poisoning Caused by Damaged Pipelines, Sindhikela, Bolangir, Orissa, India, 2008

**DOI:** 10.1155/2009/692496

**Published:** 2009-07-01

**Authors:** Manjubala Panda, Yvan J. Hutin, Vidya Ramachandran, Manoj Murhekar

**Affiliations:** ^1^Field Epidemiology Training Programme, National Institute of Epidemiology (NIE), Indian Council of Medical Research, Chennai, Tamil Nadu 600077, India; ^2^WHO India Country Office, Nirman Bhavan, Maulana Azad Road, New Delhi 110011, India

## Abstract

*Introduction*. We investigated a cluster of pesticide poisoning in Orissa. *Methods*. We searched the village for cases of vomiting and sweating on 2 February 2008. We described the outbreak by time, place, and person. We compared cases with controls. *Results*. We identified 65 cases (two deaths; attack rate: 12 per 1000; case fatality: 3%). The epidemic curve suggested a point source outbreak, and cases clustered close to a roadside eatery. Consumption of water from a specific source (odds ratio [OR]: 35, confidence interval [CI]: 13–93) and eating in the eatery (OR: 2.3, CI: 1.1–4.7) was associated with illness. On 31 January 2008, villagers had used pesticides to kill street dogs and had discarded leftovers in the drains. Damaged pipelines located beneath and supplying water may have aspirated the pesticide during the nocturnal negative pressure phase and rinsed it off the next morning in the water supply. *Conclusions*. Innapropriate use of pesticides contaminated the water supply and caused this outbreak. Education programs and regulations need to be combined to ensure a safer use of pesticides in India.

## 1. Introduction

Pesticides enhance economic potential through increased food and fiber production as well as through control of vector-borne diseases. However, they may also result in serious negative environmental and health implications [1]. Organophosphorus compounds have largely been used as pesticides in many parts of the world [1]. They are readily available because of insufficient regulations to control their sale [1]. This ease of availability has resulted in a gradual increase in accidental and suicidal poisoning [1–4]. Globally, each year, poisoning due to organophosphorus compounds is responsible for approximately three million episodes that result in nearly 200 000 deaths [5]. The vast majority of these occur in developing countries like India, as a result of fatal pesticide self-poisoning [6–8]. However, developing countries account for only 20% of global pesticide use [5]. 

On the morning of 2 February 2008, residents of Sindhikela village, Bolangir district, in the Indian state of Orissa were admitted to the hospital with complaints that included vomiting, profuse sweating and fits. Some were in serious condition. This created agitation and civil unrest in the population. Sindhikela is a small village with a poor rural population, where people sometimes do not have the resources to cook themselves breakfast: they eat a snack on the street instead. As a response to the alert, the Additional District Medical Officer (Public Health) and a graduate from the Indian Field Epidemiology Training Programme (FETP) proceeded to the village immediately with the district task force to identify for the cause of the outbreak and ensure preventive and control measures. 

## 2. Method

### 2.1. Descriptive Epidemiology

A physician examined all patients. We defined a case as occurrence of vomiting and profuse sweating between 6:00 AM and 8:00 AM on 2 February 2008 in a resident of Sindhikela. We searched for cases among patients attending the hospital and in the population. We prepared a list of all cases. We plotted an epidemic curve, drew a map of the residence of the case patients, and calculated age and sex specific attack rates to describe the outbreak by time, place, and person.

### 2.2. Analytical Epidemiology

To identify risk factors we conducted a case control study. We included all cases as defined above and recruited controls as the healthy neighbors of case patients. We collected information from the case patients and the control subjects on symptoms, date and time of onset, source of food taken in the last 24 hours and drinking water source. We analyzed the data with the help of Epi info 2005 (CDC). We compared the frequency of exposure variables between cases and controls through odds ratio with 95% confidence intervals.

### 2.3. Laboratory Studies

We collected residual specimens of food from a roadside eatery that we suspected as a possible source and sent them to a laboratory. We collected water specimens from different drinking water sources and sent them for chemical analysis to the public analyst, State Surveillance Cell, Bhubaneswar, the state capital. We collected vomitus from few case patients and sent those for examination.

### 2.4. Environmental Investigations

We interviewed the local population regarding the event. They reported that many affected had eaten in a particular roadside eatery. We visited the eatery and interviewed the owner. We traced back the drinking water source and examined the water supply system.

## 3. Result

### 3.1. Descriptive Epidemiology

**Figure 1:**
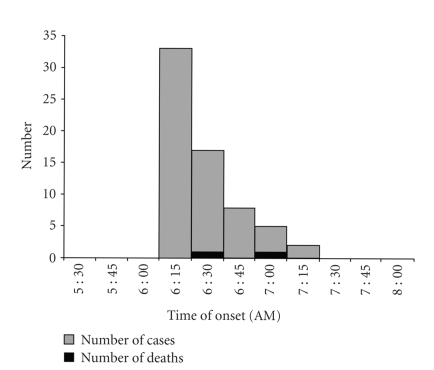
Cases of organophosphorous poisoning by time of onset at Sindhikela, Bolangir, Orissa, India, 2 February 2008.

**Figure 2:**
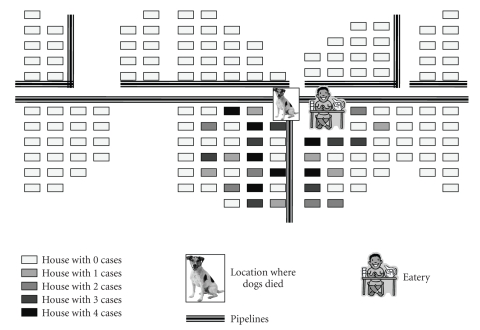
Organophosphorous poisoning cases in Sindhikela, Bolangir, Orissa, India, 2008.

**Figure 3:**
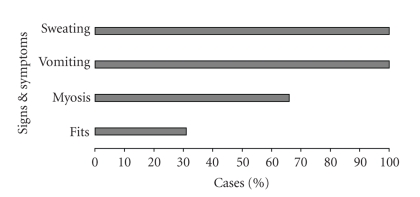
Frequency of signs and symptoms among patients with organophosphorous poisoning, Sindhikela, Bolangir, Orissa, India, 2008.

Sindhikela is located 100 km away from Bolangir (2008 population: 5263; Male/Female: 2680 : 2613). We confirmed the occurrence of an outbreak as 65 cases and two deaths were reported within an hour in the context of a background rate of less than one hospitalization per month (overall attack rate: 12 per 1000, case fatality: 3%). The distribution of cases over time (Figure 1) pointed to an initial peak of 33 cases between 6:15 AM and 6:30 AM followed by a decline and two last cases at 7:15 AM, suggesting a point source outbreak. The spot map (Figure 2) indicated that there was a clustering of cases in Harijanpada and Hatpada, two adjacent localities of the village. The attack rate per 1000 was the highest among the 5 to 8 years of age and among males (Table 1). Vomiting and sweating were present in all cases (as per our definition); whereas 77% had myosis and 31% convulsions (Figure 3). The proportion of case patients with convulsions decreased with age, from 83% (10/12) among children 0–4 years of age, to 60% (6/10) among 5–8 children years of age, to 17% (2/12) among children 9–13 years of age to 6% (2/31) among persons 14 years of age and older (*P* < .001, Chi square, 3 degrees of freedom).

### 3.2. Analytical Epidemiology

The point source pattern of the outbreak and the clustering in a specific location close to a roadside eatery led us to suspect a mixed foodborne and waterborne origin. We included all 65 patients cases and 65 healthy neighbors as controls in our case control analysis. Cases and controls did not differ by sex. However, cases were more likely to be children 0–13 years (OR: 3, 95% confidence interval [C.I.]: 1.4–6.2, Table 2). Consumption of water from a specific source (OR: 35, C.I.: 13–93) and eating at the roadside eatery (OR: 2.3, C.I.: 1.1–4.7) was associated with illness (Table 2). 48 cases and 36 controls had eaten in that roadside eatery that morning (the eatery was popular, even among small children). In an analysis restricted to those who had eaten in the eatery, cases were more likely to have eaten pakoda with a water-based chutney preparation (OR: 3.9, C.I.: 1.4–11).

### 3.3. Laboratory Studies

Neither the food specimens nor the vomitus collected from three case patients grew any organism on culture.

### 3.4. Environmental Investigations

On 31 January 2008, some villagers had used a pesticide (Folidol EC 50, an emulsifiable concentrate) to kill street dogs. The pesticide was given in a container made of leaves. Three dogs died on the next day. The containers with the residual insecticide were thrown in the drains near Harijanpada. Pipelines supplying drinking water that were damaged at many places were located beneath the drain. These pipelines supplied drinking water twice a day, while during the rest of the time, they had a negative pressure. On 2 February 2008 early in the morning (6 AM), when the first phase of drinking water was supplied to Harijanpada, it may have rinsed off pesticide that had been aspirated in the pipelines during the nocturnal phase of negative pressure. The owner of the small roadside eatery near Harijanpada collected water from that particular point to prepared pakoda and the water-based chutney.

## 4. Discussion

The active ingredient of Folidol is Methyl Parathion, an organophosphorous compound. When ingested, it acts by inhibiting enzyme acetylcholinesterase in the body. This leads to accumulation of acetylcholine resulting in over stimulation of muscarinic and nicotinic receptors of skeletal muscles. This causes a syndrome including vomiting, sweating, and convulsion. The case patients during this outbreak had a typical syndrome of organophosphorous poisoning. However, we could not do any serological test to exclude another etiology nor estimate the serum cholinesterase and red blood cell cholinesterase levels. Further, autopsy could not be practiced on the two deceased case-patients because they died on their way to the hospital. We treated patients at the Sindhikela community health center, referred serious cases to subdivisional hospital, Titilagarh, and to a medical college hospital in Burla. We treated patients with intravenous Atropine, intravenous fluids, and other supportive measures [9].

The population of the village frequently used Folidol to kill rats and street dogs, an inappropriate indication. After the dogs were killed, the containers with the residual insecticide could have contaminated the drinking water as the damaged pipe lines were going right beneath the drains and presented negative pressure. The actual product that was used, Folidol EC 50, is an emulsifiable concentrate, a WHO class I product. Those are actually banned for use in India (the villagers reported that they used an old stock procured before the ban). An accidental poisoning of a less potent organophosphorous (WHO class II or III) would have been unlikely to have lead to this many casualties.

In addition to deliberate self-poisoning with pesticide, the burden of accidental poisoning with organophosphorous is unclear, because these episodes are not systematically reported. As an example, in 2006, in the same area of Bolangir, in the state of Orissa, we investigated a cluster of 33 cases of organophosphorous poisoning secondary to inhalation of Themit. The cluster occurred among agricultural workers who were planting in a paddy field that had been recently sprayed. Because of the nature of their activity, they were working with their face less than a meter away from the ground. If it were not for the presence of an epidemiologist from the Field Epidemiology Training Programme (FETP) of India posted in the district (an unusual event given the number of trainees and the size of the country), this cluster would not have been reported. 

We communicated our findings to public health decision makers and local authorities. We stopped supplying the suspected contaminated drinking water and provided alternate drinking water through tankers. We discussed with villagers the issue of safe drinking water and proper handling of pesticides (including abstaining from inappropriate indication such as use to kill dogs). We recommended replacing and relining the pipeline. In the longer term, systematic epidemiological investigations such as the one we report must be conducted for clusters of toxicoses of unknown origin [10]. Facilities to diagnose organophosphorous poisoning in relevant health care facilities and systematic reporting of clusters would also provide additional capacity to document and prevent such occurrences.

## Figures and Tables

**Table 1:** Incidence of organophosphorous poisoning by age and sex, Sindhikela, Bolangir, Orissa, India, 2008.

Demographic characteristics		Cases	2008 population	Attack rate per 1000
Age	0–4	12	524	23
	5–8	13	510	26
	9–13	11	675	16
	14+	29	3554	8

Sex	Male	42	2650	16
	Female	23	2613	9

Total		65	5263	12

**Table 2:** Characteristics of organophosphorous poisoning cases and controls, Sindhikela, Bolangir, Orissa, India, 2008.

Characteristics	Cases			Controls			OR	Confidence interval
	Number	Total	%	Number	Total	%		
Age (0–13 years)	36	65	55	19	65	29	3.0	1.4–6.2
Sex (Male)	42	65	65	40	65	61	1.1	0.5–2.3
Drinking supply water	54	65	83	8	65	12	35	13–93

Roadside eatery attendance								
Eating any foot item	48	65	74	36	65	55	2.3	1.1–4.7
Eating pakoda alone^(1)^	27	48	56	30	36	83	0.3	0.1–0.7
Eating pakoda & chutney^(1)^	21	48	44	6	36	17	3.9	1.4–11

^(1)^Analysis restricted to the 48 case patients and the 36 control subjects who ate at the roadside eatery.
